# Emergency First Responders’ Misconceptions about Suicide: A Descriptive Study

**DOI:** 10.3390/nursrep14020060

**Published:** 2024-03-28

**Authors:** Elena Victoria Ayala Romera, Rosa María Sánchez Santos, Giulio Fenzi, Juan Antonio García Méndez, Jose Luis Díaz Agea

**Affiliations:** 1 Faculty of Nursing, Catholic University of Murcia (UCAM), 30107 Murcia, Spain; evayala@alu.ucam.edu (E.V.A.R.); jagmendez@ucam.edu (J.A.G.M.); 2 Specialist Mental Health Nurse, SAMU 061 Balearic Island, 07014 Palma de Mallorca, Spain; rosamaria.sanchez@061balears.es; 3 Faculty of Nursing, University of Murcia, Campus de Ciencias de la Salud, Edificio LAIB/DEPARTAMENTAL, El Palmar-Murcia, 30120 Murcia, Spain; agea@um.es

**Keywords:** nursing, mental health nursing, first responders, suicide

## Abstract

Background: In 2022, suicide was the second leading cause of external death in Spain (the first among young people aged 15–29 years). This study aims to analyze the presence of myths among emergency first responders and identify the most prevalent false beliefs among them. Methods: The research is a observational and descriptive study carried out using a questionnaire composed of a total of 25 myths, with the response options being true or false. A total of 543 professionals took part in the study. All of them could intervene before, during, and after a suicide attempt. Results: The main finding of the study is that more than 50% of the participants accept as true the statement "There are more serious and less serious problems", underlining the idea that caring for patients could be related to the importance the health professional gives to the patients’ problem. Myths such as “The suicidal person wants to die” and “The suicidal person is determined to die” are also evident. Conclusion: The subjective thought the first responder has about suicide could affect their acts, and there is a need to train first responders in suicidal behavior to be able to create an adequate approach.

## 1. Introduction

Suicide is defined as the voluntary and deliberate act of ending one’s own life. It is carried out by the individual, who aims for a lethal outcome. There are two inherent characteristics of suicide: lethality and intentionality. The first term refers to the result, which cannot be anything other than the death of the person, while the second refers to the decision of the subject [1].

Suicide has had different connotations and attributions according to the dogma and the predominant paradigm at each historical moment. Throughout history there can be found many connotations, some even being antagonistic to each other [2]. Mentions of suicide are already found in some ancient Greek works. It was seen as being part of life if the subject provided arguments for carrying it out. Most authors relate it to the power of the gods. It is also mentioned in works by Plato, Socrates, and Aristotle. The latter refers to suicide as an act of cowardice and dishonor that not only has repercussions on a personal level, but also on the society to which the person belongs. It was only during the 19th century that the first attributions of suicide to mental illness began to appear. At the beginning of the 20th century, a movement arose, supported by psychoanalysts, in which suicide was connoted as the poor control of impulses such as violence and death [3].

Between the 20th and 21st century, awareness of the importance of suicide has been growing. Research on suicide has increased considerably in the last 40 years. It accounts for 10% of published studies in psychiatry. Despite the increase in the number of studies, there have not been significant changes or improvements. In the 21st century, the total number of suicides has increased by 20.75%. More than 80,000 people died by suicide [4].

Provisional data from the Spanish Foundation for Suicide Prevention (FSME) published in 2023 and referring to data collected during the year 2022 [5], show a total of 4097 suicides compared to the 4003 suicides recorded during the year 2021 [6]. Spain, in comparison with other countries, is considered to have a low suicide rate (7.6 per 100,000 inhabitants). Worldwide, the countries with the highest number of suicides per 100,000 inhabitants are Korea (24.1%), Lithuania (18.5), and Slovenia (15.7) [7]. These figures could be altered, as the certification of a death by suicide is complex, due to the difficulty of proving the intentionality of the subject. Furthermore, it should be considered that there has been a tendency throughout history to conceal suicide either because of the tragic connotations of the situation or out of respect for the customs of the environment [2].

Those who find themselves in the position of caring for a person with suicidal behavior face numerous psychological, emotional, and systemic difficulties. Firstly, there are the caregivers (whether family members or not). Among these people, there is the negative perception of the caregiver himself/herself, who does not feel any benefit from caring for the suicidal patient and experiences feelings of helplessness [8]. The most direct causes of this feeling can be found in healthcare systems and the professionals who work in them. Doctors and nurses have a negative perception of a suicidal patient admitted to the emergency department, who is perceived as “problematic” or as a person who “attracts attention” [9,10]. Even more difficulties are encountered by professionals caring for patients with repeated suicide attempts. They feel powerless and see no benefit in their care themselves [11]. Negative thoughts on the part of doctors and nurses are associated with a lack of knowledge and inexperience, and the emotional response of healthcare workers affects the thought processes of patients [12]. Decreased attention and recognition by healthcare workers may be a factor in repeated suicide attempts and in the quality of care given by caregivers [13].

To approach the problem, it is advisable to improve the clinical understanding and awareness of common myths surrounding suicide being barriers to care [14]. This study was performed to address suicide prevention in a comprehensive manner and to gain an in-depth understanding of such a complex problem. The aim of this study is to analyze the myths about suicide among emergency first responders and the groups directly involved in the care of suicidal patients.

## 2. Materials and Methods

### 2.1. Design and Settings

This is an observational and descriptive study conducted in the Balearic Islands (Spain) between November 2021 and March 2023. The data come from the completion of a self-administered survey, based on the dichotomous choice of the participants regarding the veracity of different myths about suicide. Qualitative or dichotomous categorical variables were used to conduct the research.

### 2.2. Participants

The participants were professionals from different emergency services facing the possibility of having to actively intervene in suicidal behavior. Convenience sampling was carried out using a non-probabilistic method. All the selected participants were active workers, working in the Balearic Islands at the time of the study. They included men and women aged between 18 and 65 years old. A total of 543 people took part in this research study: 378 of those belong to the local police; 125 to the SAMU 061 emergency medical staff (including doctors, nurses, and emergency medical technicians); and 40 were social workers and family workers. The inclusion and exclusion criteria are shown in Table 1.

### 2.3. Procedure

The information was collected in person on different dates from November 2021 to December 2022. The data analysis phase was carried out between 7 February 2023 and 16 March 2023.

Data collection was carried out in the Balearic Islands, in person at different locations of the SAMU 061 management, local police classrooms, and training classrooms in Ibiza and Menorca. Participation was voluntary. The methodology involved was the creation of a questionnaire comprising 25 myths about suicide (Table 2).

The questionnaire offered a dichotomous and nominal response format (true or false). The sequence of the myths was not predetermined. If respondents selected “True”, it meant that what was presented as a myth in the questionnaire was a real credence in their belief. In opposition, if participants selected “False”, it meant they disagreed with the sentence and recognized it as something not real. Doing so, they accepted it as a valid myth.

To ensure the questionnaire’s validity, the main myths about suicide were identified based on existing scientific evidence on the topic [14,15,16,17,18]. They were then organized into 5 categories to better understand their contents and meanings (Table 3).

This content was then used to develop the questionnaire. Additionally, three specialists in the field, including two with PhDs in Psychology and a clinical psychologist, reviewed the questionnaire for its relevance and accuracy. All three reviewers unanimously agreed on its validity, resulting in a content validity index of 1 (CVI = 1). In order to assess the internal consistency of the instrument, Cronbach’s alpha coefficient was calculated, yielding a result of over 0.7, indicating satisfactory internal reliability and supporting the robustness of the measurements. The final questionnaire was then distributed physically among the participants.

Following data collection, one researcher (E.V.A.R.) conducted the initial analysis using a spreadsheet program. Subsequently, two other researchers (R.M.S.S.; J.A.G.M.) reviewed the analysis for accuracy.

### 2.4. Data Analysis

After collection, the data underwent variable encoding (0 = True and 1 = False) and were categorized according to the study participants (1 = local police LP; 4 = healthcare workers HCW; 3 = social workers SW). Each participant was assigned a unique ID number in consecutive order. Likewise, the myths were sequentially numbered from P1 to P25. Subsequently, a spreadsheet program (Excel 2021^®^) was employed to perform the tabulation of the various variables for the study.

The data analysis is performed following three-step calculations across the entire participant pool: the absolute frequency, relative frequency, and percentage. Firstly, the absolute frequency is computed, aiming to ascertain the frequency of the occurrence of identical conditions among participants. Specifically, this was to do with the occurrence of true, false, and blank responses to each myth. Subsequently, relative frequency is determined by dividing each absolute frequency by the total number of respondents to those myths. The outcomes are also conveyed as percentages, derived from multiplying the relative frequencies by 100. After gaining first a general picture, these are stratified based on professional categories. The aim is to ascertain whether the aggregate findings align with those obtained individually within each subgroup: local police, social workers, and healthcare personnel. These data are utilized to visually represent the diverse responses obtained and to scrutinize the parallels and discrepancies among them.

### 2.5. Ethical Consideration

The study was conducted according to the guidelines of the Declaration of Helsinki. Informed consent was obtained from all subjects involved in the study. We followed the guidelines of the American Psychological Association (APA) regarding ethical issues (Sections 3 and 8 of the Ethical Principles of Psychologists and Code of Conduct) [19]. In this sense, the APA does not consider it necessary to include an ethics committee when there is no risk related to anonymity or confidentiality in a study. The descriptive, observational study we conducted did not require any interventions that could have consequences for the study participants. During the process, no manipulation of variables was carried out. Participants were invited to fill in the questionnaire anonymously and individually to promote their freedom of response without being conditioned by the subsequent analysis of the data and so as not to coerce their response.

## 3. Results

The analysis of the data from the questionnaire shows the existence of widespread myths among first responders and professionals involved in the care of suicidal patients (Table 4).

“There are serious and less serious problems”. More than half of the participants affirmed this myth as being true, more specifically 56.72% compared to 42.33% who marked it as false. In total, 0.92% did not provide any answer. It thus becomes the most widespread cliché among the professionals involved in this research study.

“The suicidal person wants to die”. This myth was the second most widespread (affirmed by 42.54%) with similar values to the third: “The suicidal person is determined to die” (41.25%). Both myths were related in their results and meaning, identifying death as the person’s ultimate goal.

Next come the myths “Those who want to kill themselves do not say so”, with a total of 39.41% of statements, and “Those who say so do not do it”. Both identified an existing prejudice about the communication of suicide.

Among the results obtained, myths related to the socio-economic situation of the patient stand out. The idea that “Suicide is more common among the poor” (12.84%) was more widespread than “Suicide is more common among the rich” (8.28%). Opposing ideas about the perception of the personality of the suicidal patient were also noted. The idea that “The person who attempts suicide is brave” (8.28%) was higher than “The person who attempts suicide is a coward” (4.05%).

The statement “The person who recovers from a suicidal crisis is in no danger of relapse”, marked as affirmative by 2.20% of those involved, was recognized as false by the majority of participants, and it was the second least prevalent myth. Most of the carers knew a suicidal person could re-attempt it.

The myth “Only old people commit suicide” deserved special mention, as the results obtained here were exceptional: 100% of the participants recognized this statement as false, confirming its recognition as a myth.

**Figure 1:**
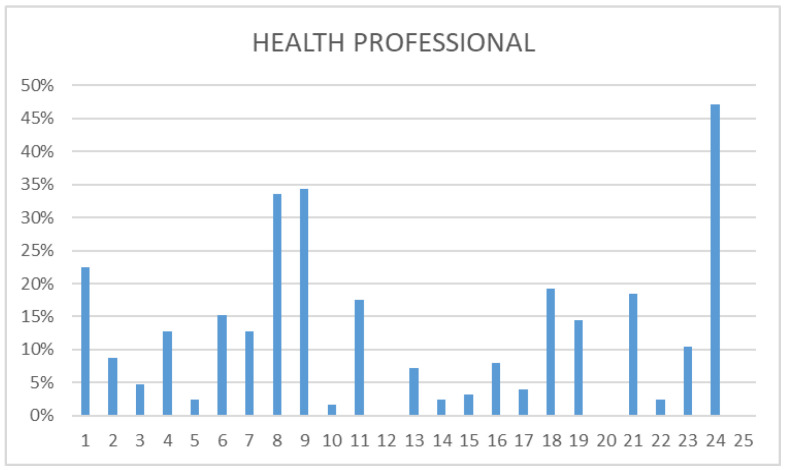
Health professionals’ questionnaire. This figure illustrates the distribution of myths among healthcare professionals (physicians, nurses, and emergency technicians) from the 061 service in Mallorca, Menorca, and Ibiza. Four myths (1, 8, 9, 24) were predominantly observed, while three were acknowledged as true myths (12, 20, 25).

**Figure 2:**
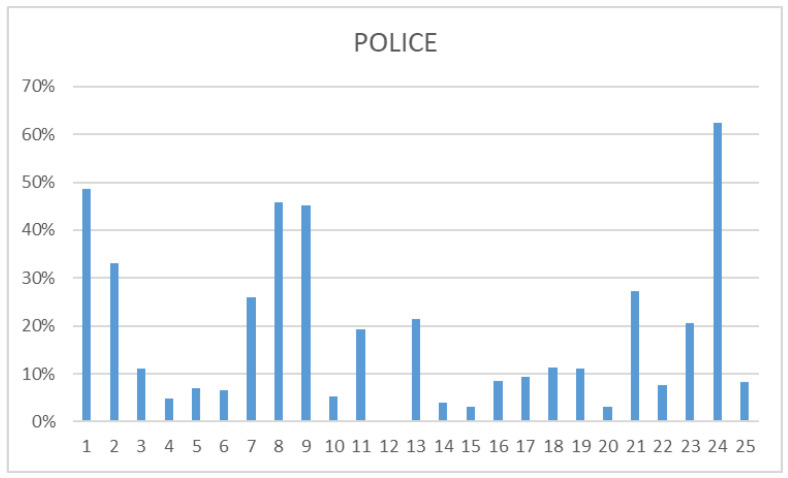
Police questionnaire. This figure illustrates the distribution of myths among the police force in Palma. Four myths (1, 8, 9, 24) were predominantly observed, while only one was recognized as a true myth (12).

**Figure 3:**
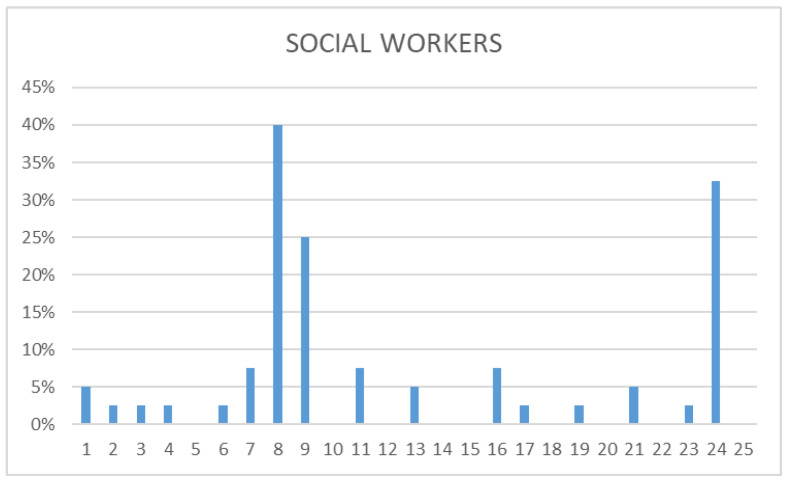
Social workers’ questionnaire. This figure shows the distribution of myths among social workers in Mallorca. Three myths (8, 9, 24) were predominantly observed, while a total of nine were recognized as true myths (5, 10, 12, 14, 15, 18, 20, 22, and 25).

The independent analysis of the data, differentiating between the different participating groups (health professionals, police, and social workers) was carried out. All three groups concurred in identifying as prevalent the following: “There are serious and less serious problems”, “The suicidal person wants to die”, and “The suicidal person is determined to die”. While social workers’ responses tended to focus more on these three myths, police and health workers provided more varied answers, acknowledging a broader range of myths. Some of the most frequently mentioned myths by police and health professionals fell into the category of “attitude”. In fact, most of them believed in myth 1 alongside myths 8 and 9. This contrasted with social workers, who mostly recognized sentence 1 as a myth. Health professionals identified three phrases as real myths: “Only old people commit suicide”, “A person who recovers from a suicidal crisis is in no danger of relapse”, and “Suicide is a crime”, whereas police only recognized the first. Social workers demonstrated a heightened awareness of myths, identifying a total of nine: 5, 10, 12, 14, 15, 18, 20, 22, and 25. This description is made based on data that can be found in the following Figure 1, Figure 2 and Figure 3.

This finding gained significance when considering the professional roles of each group. Both health personnel and local police were considered first responders. The first linked with a better knowledge of health problems while the second focused on supporting the subjects and researching victims’ information. On the other hand, social workers typically engaged in more extensive conversations with patients and may or may not initially respond.

## 4. Discussion

Our study highlights that many suicide myths are considered valid by first-line caregivers, impacting the initial stages of care provision. The intricate management of suicide cases, coupled with misconceptions about such behavior, presents challenges for out-of-hospital healthcare and emergency responders. The circumstances surrounding suicide attempts vary widely, sometimes masking the true intention behind activations. Ambulance services and emergency departments are thus overlooking opportunities to offer improved care to this population, leading to potentially avoidable mortality, morbidity, and service burdens. Individuals facing a mental health crisis often receive care of varying quality compared to those experiencing physical health crises. The admission of these patients to emergency services may be viewed unfavorably, with treatment strictly confined to their physical condition, while subsequent care provision or psychiatric investigations may be neglected or ignored [20].

Suicidal behavior has different manifestations, so there are variables unknown to the professional. This is why this type of intervention involves an extra emotional component (fear, rejection, misunderstanding, taboo, myths, etc.) affecting the different professional, emotional, and legal spheres. Dealing with these patients can be very complex [13]. A suicide attempt is an emergency, and as such requires immediate decisions to be taken to safeguard the threatened life. In addition, actions need to be taken to reduce the risks to the safety of the victim and the professionals involved [21]. Emergency professionals are used to assisting patients who are struggling to live, and this issue breaks the mental model of the responders and can have a profound impact on them if the suicide is completed and, above all, witnessed [22].The role of these professionals becomes crucial in treatment as well as prevention to provide a good quality of care from the first second of attendance [23]. This aspect is in line with the World Health Organization (WHO), which makes it a priority to enhance nations’ capacity to develop and evaluate suicide-prevention plans and policies, with actions tailored to the specific needs of the countries. Specifically, it urges the implementation of prevention measures based on scientific evidence [24].

As mentioned earlier, this study aims to identify prevailing myths and misconceptions about suicide among emergency professionals and first responders. The results reveal the persistence of such myths, underscoring the need to debunk them and establish goals for future educational initiatives [14].

Performing a more rigorous analysis of the myths, we could observe the following:

“There are serious and less serious problems”: this was the most prevalent in the results. This underlines the different importance given to the suicide depending on the person who cares for those patients and the person’s perceptions of reality and the problem he/she is facing [25]. This myth requires thorough examination, as problems cannot simply be assessed or classified based on severity alone; there lacks an objective method capable of accurately measuring them. The torment experienced by the individual is closely connected to how they see these problems. Often, it is driven by feelings of hopelessness and frustration. It is crucial to remember that suicide is a complex action stemming from internal experiences, and the connection between suffering and its triggers is not always direct [1]. So, simplifying it as just a reaction to external factors does not capture its complexity. Understanding how internal struggles and outside pressures interact is vital for helping those with suicidal thoughts and preventing tragedies. It shows the importance of a comprehensive approach to mental health care, including emotional support and practical help tailored to each person’s needs [2].

“The suicidal person wants to die” and “The suicidal person is determined to die”. These myths rank as the second most prevalent. According to current scientific evidence, there is often ambivalence about death in most cases. Individuals may not be determined to die or have a desire for death but rather seek an end to their suffering. It is important to recognize this perspective in understanding suicidal thoughts. By acknowledging the complexity of these feelings, we can better address the underlying issues and provide appropriate support [26]. Understanding the ambivalence towards death underscores the need for compassionate and comprehensive interventions tailored to individuals’ unique experiences [27]. Suicide usually goes through three phases: during the first phase, the person considers suicide as a method of solving problems, then there is an ambivalence between the positive and negative aspects. If the negative aspects outweigh the positive ones, the third phase is the stage where the decision to take one’s own life is firm. Intervention at this stage would be focused on generating this ambivalence again to assess aspects that the subject did not contemplate [1].

“Those who want to kill themselves don’t say so” and “Those who say so don’t do it” are the fourth and fifth most prevalent. Both myths minimize the perceived threat of suicide for the person on the receiving end. Between 8 and 9 people out of 10 who have tried had stated their intention to take their own life directly, prior to carrying out this action. And one of them expressed it through indirect signs [28]. Communication can take place verbally, directly, or indirectly (behavioral manifestations: they do things they do not usually do, they stop doing things they were doing), but the person can also give indications of their intentions through actions, such as giving away valuable objects, saying goodbye to family and friends, putting their things in order, making a will (“closing behaviors”), or acquiring material to be able to go ahead with the act and carry out the suicide attempt. The above is behavioral. There can also be written manifestations (10% farewell notes) and they can also do it through social media (the ways to express it are verbal, written, behavioral, and on social media).

The results and consequent analysis bring out a very controversial issue. These findings indicate an automatic response in the form of myths. They shape the beliefs and prejudices of first responders, influencing their attitudes towards suicide attempts. This reveals a pattern where beliefs and preconceptions can influence how emergency professionals perceive and respond to individuals in crisis. Understanding the impact of these myths is crucial in addressing the stigma and barriers surrounding mental health emergencies. By recognizing and challenging these misconceptions, we can promote more compassionate and effective responses to individuals in need of support during suicidal crises. This brings us closer to the studies by Libet and Haynes [29,30], which highlight the unconscious nature of our decisions.

To respond to the myths about suicide, experiential and reflective training is necessary [31]. It allows for levels of deep reflection to dismantle prevailing mental models (beliefs and myths) through different methods such as debriefings, simulations, role playing, or focal groups [32,33,34]. The only immediate requirement to achieve this level of depth is the creation of safe spaces in the group that is receiving the training. For this, there are methodology, such as the SC 3.0 (Clinical Simulation 3.0) [35], that focus on building an atmosphere of trust through different activities. Establishing such an environment could aid in developing training programs focused on designing simulation scenarios that simulate situations involving patients at risk of suicide and the care provided by first responders. Through these simulations, emergency responders can engage in various scenarios and, during the debriefing sessions, reflect on the learning outcomes and consider whether their actions during the simulation were influenced by preconceptions, biases, or myths. This reflective approach fosters a deeper understanding of their responses and enables them to refine their practices for real-life situations [32].

### Limitations

This research would gain from additional statistical analyses, exploring correlations between the acceptance of myths and socio-demographic variables. The questionnaire could have more indications of reliability, which would increase the validity of the study. It is important to note that the data solely reflect the study participants and may not be generalized to the wider population. Future research endeavors could focus on addressing these limitations and exploring other potential areas of improvement.

## 5. Conclusions

This study analyzed the validation of myths (giving as valid statements considered to be myths about suicide) among first responders in the care of suicidal patients.

The main finding shows that more than half of the respondents make a quick judgement about the appropriateness of suicidal behavior, as the most accepted myth was that “There are serious and less serious problems”. This shows that first responders may judge the degree of suffering of people at risk of suicide based on their own mental models and perception of suffering.

The attitude of the first responder in accessing a person at risk of suicide is decisive for the patient’s safety and the efficiency of the therapeutic approach. If a first responder considers that the situation does not justify the suicidal risk, the therapeutic approach will certainly not be excellent. To rush to a judgement in this situation, whatever the decision, is a mistake; however, it is part of the automated human response. Being aware of the existence of this risk should be the first step in working on suicide training.

Further studies are needed to find out the impact that false myths about suicide have on initial patient care. This preliminary study sheds light on possible misconceptions about suicide attempts among health professionals.

## Figures and Tables

**Table 1:** Inclusion–exclusion criteria.

Inclusion Criteria	Exclusion Criteria
Voluntary participation	Failure to complete the questionnaire in full
Being in active service	Leaving during the session
Doctors, nurses, emergency technicians from ambulance service of Mallorca, Menorca, Ibiza	Healthcare personnel working in a hospital environment
Local police workers from Palma	Professionals who subsequently provide the training
Social workers from Mallorca	
Completion of the questionnaire in person	

**Table 2:** Script of myths that make up the questionnaire.

Myth Number	Myth
1	Those who want to kill themselves do not say so
2	Those who say so do not do it
3	Everyone who die by suicide are mentally ill
4	Suicide is inherited
5	The suicidal person just wants attention
6	Suicide is more common among the rich
7	Talking about suicide with a person who is at risk may encourage them to commit suicide
8	The suicidal person wants to die
9	The suicidal person is determined to die
10	The suicide attempter is a coward
11	Suicide is a normal reaction to negative experiences
12	Only old people commit suicide
13	Suicide is impulsive
14	If a suicidal person is challenged, he does not attempt it
15	Interviewing family members is a breach of confidentiality
16	A suicide attempter is a brave man
17	The media cannot contribute to suicide prevention
18	Suicide is more common among the poor
19	Once suicidal, one is never suicidal again
20	A person who recovers from a suicidal crisis is in no danger of relapse
21	Most suicides happen suddenly and without warning
22	Only psychiatric professionals can prevent suicide
23	People displaying suicidal behavior are dangerous
24	There are serious and less serious problems
25	Suicide is a crime

**Table 3:** Categories of the myths used in the questionnaire.

Category	Definition	Miths
Attitude	Belief related to some attitude of the patient displaying suicidal behaviors.	1; 8; 9; 13
Behavior	Belief related to the behavior of/to the patient displaying suicidal behaviors or his/her family members.	2; 7; 14; 15
Trait	Myths related to traits of the patient displaying suicidal behaviors. Belief related to some personal trait of the patient.	3; 6; 10; 12; 16; 18; 19; 20; 23
Environment	Myths related to the environment of the patient displaying suicidal behaviors. Belief related to some aspect of their environment.	4; 5; 17; 22; 24
Act	Myths related to the act of suicidal behavior. Belief related to the act itself.	11; 21; 25

**Table 4:** Representation of the percentages of responses out of the total number of participants.

Myth Number	True		False		Not Answered	
	Frequency	Percentage	Frequency	Percentage	Frequency	Percentage
1	214	39.41%	325	59.86%	4	0.73%
2	137	25.23%	402	74.04%	4	0.73%
3	49	9.02%	492	90.62%	2	0.36%
4	35	6.44%	507	93.38%	1	0.18%
5	30	5.66%	512	94.16%	1	0.18%
6	45	8.28%	494	90.99%	4	0.73%
7	117	21.54%	423	77.91%	3	0.55%
8	231	42.54%	305	56.15%	7	1.31%
9	224	41.25%	311	57.28%	8	1.47%
10	22	4.05%	519	95.59%	2	0.36%
11	98	18.04%	441	81.23%	4	0.73%
12	0	0%	543	100%	0	0%
13	94	17.31%	442	81.38%	7	1.31%
14	18	3.31%	524	96.51%	1	0.18%
15	16	2.94%	515	94.86%	12	2.20%
16	45	8.28%	486	89.52%	12	2.20%
17	41	7.55%	497	91.53%	5	0.92%
18	69	12.83%	469	86.25%	5	0.92%
19	61	11.20%	474	87.33%	8	1.47%
20	12	2.20%	530	97.62%	1	0.18%
21	128	23.57%	406	74.78%	9	1.65%
22	32	5.89%	507	93.38%	4	0.73%
23	92	16.94%	448	82.51%	3	0.55%
24	308	56.72%	230	42.36%	5	0.92%
25	48	8.75%	485	89.28%	10	1.97%

## Data Availability

The data presented in this study are available in the Section 3 of this article.

## Public Involvement Statement

There was no public involvement in any aspect of this research.

## Guidelines and Standards Statement

This manuscript was drafted against the STROBE guidelines (The Strengthening the Reporting of Observational Studies in Epidemiology) [36] for Cross-Sectional research”.
